# The Effect of Fitness on Performance, Exertion, and Cognition During Simulated Firefighter Occupational Tasks

**DOI:** 10.3390/jfmk10020129

**Published:** 2025-04-11

**Authors:** Philip J. Agostinelli, Nicholas C. Bordonie, Braxton A. Linder, Ann M. Robbins, Parker L. Jones, Lee F. Reagan, C. Brooks Mobley, Matthew W. Miller, William M. Murrah, JoEllen M. Sefton

**Affiliations:** 1 Warrior Research Center, Auburn University, 301 Wire Road, Auburn, AL 38649, USA; philip.agostinelli@pepsico.com (P.J.A.); ncb0039@auburn.edu (N.C.B.); annrobbins0620@gmail.com (A.M.R.); plj0006@auburn.edu (P.L.J.); lreagan@uab.edu (L.F.R.); 2 Neurovascular Physiology Laboratory, Auburn University, 301 Wire Road, Auburn, AL 38649, USA; bal0039@auburn.edu; 3 Nutrabolt Applied and Molecular Physiology Lab, Auburn University, 301 Wire Road, Auburn, AL 38649, USA; moblecb@auburn.edu; 4 Performance and Exercise Psychophysiology Lab, Auburn University, 301 Wire Road, Auburn, AL 38649, USA; mwm0024@auburn.edu; 5 Department of Educational Foundations, Leadership, and Technology, Auburn University, Auburn, AL 38649, USA; wmm0017@auburn.edu

**Keywords:** training status, exercise, tactical athletes, fire services, fitness assessment, fitness screening

## Abstract

**Background:** Fitness is relevant for firefighter occupational performance, but its influence on exertion and cognition during occupational tasks remains unclear. We aim to determine fitness’s influence on performance, exertion, and cognition during simulated firefighter occupational tasks. **Methods:** Two baseline fitness assessments occurred to measure aerobic fitness, strength, power, and body composition in 33 non-firefighter participants (females/males: 15/18; 25.18 ± 4.06 years; 174.01 ± 9.77 cm; 75.94 ± 13.18 kg). A third visit involved participants completing an occupational task assessment (OTA; four rounds of deadlifts, sandbag carries, and a cognitive assessment at 35 °C/50% humidity) to the best of their ability. Multiple linear regression models, including strength and power, aerobic capacity, and body composition, were used to predict OTA performance and exertion. Our outcomes included time to complete, exertion, and cognitive performance during the OTA. **Results:** The model predicted OTA time, RPE, skin temperature, and blood lactate (*p*s < 0.02), but not core temperature (*p* > 0.24). The model did not predict cognitive errors (*p*s > 0.06). **Conclusions:** These metrics of fitness provide meaningful predictive insight into firefighters’ occupational readiness during simulated occupational tasks.

## 1. Introduction

Firefighting requires strength to quickly evacuate individuals as well as lift, carry, and move objects or debris [1] often ranging from 80 to 135 lbs [2]. Aerobic endurance is also required for tasks such as salvage, search, stair climbs, and shoveling [3]. These operations can result in a peak VO_2_ of 41.5 mL/kg/min or greater, heart rates of 84–100% of heart rate max, and peak lactate concentrations up to 13.2 mM [2,4]. In addition, personal protective equipment (PPE) is worn to protect firefighters from extreme heat and other dangers [5,6]. However, the insulative nature that protects the individual from the external environment also creates a significant strain on their thermoregulatory system due to the inability to evaporate sweat [7]. Additionally, this gear can impact lower-body gait mechanics [8]. This external load and the restricted movement from PPE exacerbate the stress imposed on the firefighters and limit their performance [5,8,9,10]. Added to the physical demands, firefighters are also chronically exposed to extreme environments (i.e., hot summer days or structural fires), and both impact occupational performance and physiological outcomes [11,12,13,14,15].

Impairments in cognitive function have also been observed when firefighters experience hot environments [16] and sleep deprivation [17]. Impairments while firefighting are likely a result of decreases in cerebral blood flow, as a larger proportion must be directed toward the skin in an effort to thermoregulate during heat stress [18], while sleep deprivation can result in changes in neurotransmitter receptor function [19], which may impair cognitive function. Previous research suggests improvements in cognition from acute and chronic physical exercise, causing increased cerebral blood flow, maximal oxygen consumption, and delivery of oxygen to cerebral tissue, resulting in cerebral structural changes and increased neurotransmitter levels [20,21,22,23,24]. Additionally, previous research indicates cognitive function is positively associated with physical fitness [25]. Therefore, one possible action to protect firefighters from these detrimental effects is incorporating regular exercise to increase fitness levels [26].

Previous research suggests aerobic fitness and relative lower-body muscular strength are the strongest predictors of work efficiency, which has been positively correlated with firefighter occupational task performance [3]. Specifically, a study revealed that young, non-obese, South African firefighters with good fitness and cardiovascular health were more likely to pass their physical abilities test. In this investigation, age, lean mass, body fat percent, oxygen consumption, upper- and lower-body strength, and weekly activity levels were associated with improved times on said physical abilities test [27]. The previous literature provides evidence of the relationship between fitness and occupational performance and firefighter physical ability test performance, yet few studies investigate this question while simulating environmental conditions often experienced in fire services. This is an important factor when considering that hot and humid summer climates are a frequent work condition for firefighters in many regions of the United States [28,29]. Additionally, research thus far has not interrogated fitness’ influence on cognitive performance in firefighters. Poor health outcomes (i.e., metabolic syndrome) have been shown to impair cognitive function [30] in firefighters, whereas regular exercise training has been shown to improve cognitive function [31] in general populations.

Understanding the impact fitness level has on a variety of occupationally relevant performance outcomes will help improve our understanding of what relevant assessments should be implemented by fire services to evaluate occupational readiness. Therefore, we aim to understand the influence of fitness level on relevant occupational and cognitive performance, as well as exertion during simulated occupational tasks. We hypothesized that strength, aerobic endurance, and body composition would predict occupational task performance, the level of exertion experienced, and cognitive performance during simulated occupational tasks.

## 2. Materials and Methods

### 2.1. Participants, Design, and Procedures

This study was a part of a larger multi-study project. Additional explanations of these methods have previously been submitted for publication [32,33]. Thirty-three participants (15 females, 18 males) were included in the current investigation (25.18 ± 4.06 years, 174.01 ± 9.77 cm, 75.94 ± 13.18 kg, 23.05 ± 7.42% body fat). All participants were members of the local community and were not active firefighters. To be included in this study, participants had to be between 19 and 45 years old, agree to be available for the full 5-week study timeline and study requirements, and be recreationally active (3+ days/week, 30 min/day). Participants were required to complete a general health screening and were excluded if they had a known medical condition preventing exercise, had or were rehabilitating from a musculoskeletal injury, had experienced symptoms or been prescribed treatment for asthma within the past 4 years, had a history of heart condition or high blood pressure, experienced angina during activities of daily living, physical activity, or exercise, had been prescribed medically supervised physical activity only by a primary care physician, or recently experienced a heat-related illness or injury (i.e., heat exhaustion, heat stroke, etc.). Participants completed a general health questionnaire and self-reported physical activity log to ensure they met all inclusion and exclusion criteria. Informed consent was then obtained by the lead author (P.J.A.) prior to enrollment in this study. Participants completed two baseline fitness assessments prior to an experimental trial involving an occupational task assessment (OTA) and cognitive function testing. The first visit included an assessment of body composition and aerobic fitness as outlined in Section 2.2 and Section 2.3, respectively. The second visit included an assessment of baseline cognitive function, muscular power, and muscular strength as outlined in Section 2.4, Section 2.5 and Section 2.6, respectively. A minimum of 48 h between each visit was required to reduce the risk of residual fatigue impacting subsequent sessions. Participants were asked to refrain from caffeine and exercise for 24 h leading up to all visits. For safety purposes, participants provided a urine sample to ensure proper hydration as determined by urine specific gravity (USG < 1.025). Urine samples were analyzed with a refractometer (V-Resourcing, Changsha, China) to ensure participants were adequately hydrated. Participants were encouraged to complete all testing at the same time of day and maintain a consistent diet throughout study enrollment. All study protocols were approved by the Institutional Review Board of Auburn University (protocol code #22-479 AR 2211).

### 2.2. Body Composition Assessment

After assessing hydration status, the participant’s height (cm) and weight (kg) were obtained using a digital scale and stadiometer (SECA, Hamburg, Germany). Lean body mass (kg), fat mass (kg), and percent body fat were measured with DEXA (Lunar Prodigy; GE Healthcare, Chicago, IL, USA). Participants were required to wear clothing without zippers or buttons (athletic clothing) and lie still on the DEXA with minimal movement and normal breathing during the scan (~12 min).

### 2.3. Aerobic Fitness

Following assessment of body composition, peak oxygen consumption (VO_2_peak) was assessed using a modified Bruce treadmill protocol (Woodway, Waukesha, WI, USA) involving a three-minute warm-up at 2.7 kph and 0% incline. After the warm-up, the first stage involved a speed of 2.7 kph and an incline of 5%, after which there were incremental increases in speed and incline that mimic the classic Bruce Protocol [34]. Lactate concentration was estimated during the VO_2_ peak assessment using a Nova Biomedical Lactate Plus Meter (Nova Biomedical, Waltham, MA, USA). These assessments were used as a measure of aerobic endurance capacity. During the test, indirect calorimetry with an automated open circuit system (Parvo Medics, Sandy, UT, USA) was utilized to monitor the participant’s oxygen consumption and carbon dioxide during the assessment. Before the assessment was started, calibration of the gas analyzers with standard gases (16% O_2_, 4.05% CO_2_) was completed. Additionally, the pneumotach flowmeter calibration was completed using a standard volume of air (3 L syringe). Blood lactate concentrations were measured during the final 30 s of each stage. Assessment termination occurred once participants reached volitional fatigue. VO_2_peak was determined as the maximal 30 s oxygen consumption value achieved during the test. Respiratory exchange ratio (RER > 1.15), heart rate max (within 10 bpm of age-predicted max), blood lactate concentrations (>8.0 mM), and volitional fatigue were utilized to verify a valid maximal effort was given by the participant. The Dmax fit from the Lactater package (Version 0.2.0) in R Statistical Programming Software Version 4.1.2 (RStudio, Boston, MA, USA) were used to estimate lactate threshold for each participant. Validation criteria, measurement, and quality assurance protocols have been outlined in previous publications related to this study [32].

### 2.4. Baseline Cognitive Assessment

On the second day of baseline testing, the modified Wisconsin Card Sorting Task (WCST) was completed. After 10 min rest in a quiet space within the laboratory, a baseline WCST was completed prior to all physically demanding assessments. The baseline WCST was conducted to give participants familiarity with the task, thereby minimizing associated practice effects [35]. Cognitive flexibility was chosen as the executive function domain of interest as it involves situational decision-making, utilizing various perspectives to solve problems while also incorporating the other domains of executive function (inhibitory control and working memory) [36]. Executive function is needed for firefighters to make rapid decisions based on a wide range of variables (e.g., type of emergency, environment, physical limitations of the individual being rescued) [17,37]. The current investigation used the computer-based 60-response version of the WCST. While no validation of this specific version of the test was complete, previous literature supports the sensitivity, reliability, and clinical use of shortened computerized modified-WCSTs [38,39,40]. During the baseline assessment, the task was completed in a quiet room with no potential distractors, while all OTA trials were completed in the environmental chamber. Participants sorted the cards based on three factors: color, shape, and number. The category for sorting was changed after every 10 cards and was repeated six times for a total of 60 responses. The modified 60-response version of the assessment was chosen for this study instead of the traditional version, which involves 60 correct responses with up to 128 total trials [41]. This decision was made to ensure an assessment of cognitive flexibility could be completed in a time-efficient fashion during the OTA. Researchers prompted the participants to quickly complete the assessment while minimizing errors. All additional instructions were outlined on the computer screen, and participants were told to review the rules of the WCST before taking the assessment. The computerized instructions were used to reduce the variability of instructions from individual researcher differences. Outcome measures from this assessment include total errors, perseverative errors, non-perseverative errors, and response time. Perseverative errors occur when making a sorting choice based on the previous sorting rule rather than the current rule, reflecting an inability to adapt to the changing rule set, while non-perseverative errors represent random errors. The use of this assessment has been previously outlined in an article submitted for publication as part of a separate aim from this work [33].

### 2.5. Vertical Jump (VJ)

After completing the baseline cognitive assessment, a maximal effort standing vertical jump was used to assess lower-body muscular power. Participants completed a counter-movement vertical jump on a Leonardo Mechanograph force platform (Novotec Medical GmbH, Pforzheim, Germany). Participants completed a total of three jumps with 1 min rest between attempts and were instructed to jump as high as possible. The highest of three attempts was recorded as the participant’s max vertical jump.

### 2.6. Three Repetition Maximum Lifts (3RM)

Following the vertical jump assessment, upper- and lower-body muscular strength was assessed using three repetition maximum (3RM) testing of barbell squat, bench press, and hex-bar deadlift. Criteria for a successful lift were based on the National Strength and Conditioning Association’s (NSCA) recommendation for 3RM testing [42]. Prior to starting the RE session, participants completed a structured warm-up, including a general and specific warm-up of a moderate-paced walk on a treadmill [43] and dynamic movements that mimicked and prepared the participant for the movement patterns of the acute resistance exercises, including bodyweight squats, hip rotations, and scapular protraction and retraction [43]. Participants completed warm-up sets at 50% of self-projected 1RM for 6–8 reps, 75% for 3–5 reps, and 85% for 1–3 reps. Their first attempt was then completed at 95% of self-projected 3RM with 2–5 min of rest between sets. Percentage of 1RM was based on self-reported one repetition max. If a participant was unsure of their 1RM, a certified strength and conditioning coach (P.J.A.) helped the individual estimate their 1RM based on their current training loads.

### 2.7. Occupational Task Assessment (OTA)

To control for levels of activity prior to the experimental trial, participants were seated in a quiet room for 30 min and allowed to work or browse on their laptops/phones or read a book. These tasks were based on recommendations of the local fire department’s description of activities commonly performed during firefighter downtime between occupational duties. Additionally, participants were informed to abstain from overstimulating and stressful work or activities. Upon completion of the 30 min rest period, participants were equipped with standard firefighting turnout gear and PPE as a firefighter would during a live emergency call. These items included bunker pants and a jacket, gloves, boots, and a helmet.

To begin the OTA, participants first entered the environmental chamber with conditions set to 35 °C (95 °F) and 50% humidity. Participants then completed repeated lift and carry tasks. The circuit began with 10 repeated sandbag deadlifts utilizing a Rogue Fitness^®^ sandbag (Rogue Fitness, Columbus, OH, USA) loaded to either 61.4 kg or 38.6 kg. A deadlift 3RM > 79.5 kg and a deadlift-to-body-weight ratio > 1.25 were used as criteria for the use of the 61.4 kg sandbag. This was followed by lifting an 18.2 kg Rogue Fitness^®^ sandbag (mimicking a firehose) from the floor and proceeding to carry the sandbag while on a treadmill. The carry task was continued until the participant reached 0.15 miles at a self-selected pace. The self-selected pace could be changed at any point during the task. After completing two rounds of this circuit, participants sat in a chair in the corner of the environmental chamber to complete the WCST using the same protocols as the baseline WCST (Section 2.4). Next, two additional rounds of the circuit were completed. Researchers recorded the time to complete the OTA starting when the participant approached the sandbag for the repeated deadlifts and stopping after completing the final sandbag carry. Researchers encouraged participants to complete the tasks to the best of their ability, as quickly and safely as possible. The environmental conditions were chosen based on their prevalence during firefighter emergency calls in the southeastern United States and are representative of hot summer days, outdoor emergencies, and salvage [2,16,28,44]. The load utilized for the sandbags was determined based on the frequency firefighters have to lift, drag, and carry objects of a similar size during emergency calls [2]. The Auburn City Fire Department was consulted through the study development process to ensure the OTA accurately reflected a firefighter’s job demands.

### 2.8. Physiological Monitoring and Perceived Exertion Measurements

During the OTA, an EQ02+ LifeMonitor (Equivital EQ02, Hidalgo, Pencoed, UK) was used to measure heart rate, ventilatory rate, and skin temperature and to estimate core temperature. Equivital Black Ghost software (Version 6.6.23.2766) uses a validated algorithm using heart rate to estimate core temperature [45,46,47]. Torso skin temperature is measured by this system using an infrared temperature sensor on the device in contact with the individual’s skin at the mid-axillary line of the thorax in line with the xiphoid process of the sternum. Blood lactate concentration (BLC) was measured upon arrival, after the 30 min rest period, as well as pre- and post-OTA as a measure of exercise intensity. BLC measurements were obtained using the Nova Biomedical Lactate Plus Meter (Nova Biomedical, Waltham, MA, USA) and were used for lactate threshold testing. Procedures outlined in a previous publication explain further details [32]. Rating of perceived exertion was recorded after each task of the OTA (i.e., sandbag deadlifts and sandbag carry) using the Borg 6–20 RPE scale [48].

### 2.9. Statistical Analysis

Each participant received a z-score based on baseline assessment outcomes. These assessments were then grouped based on strength (3RM bench, squat, deadlift, max vertical jump), aerobic (VO_2_peak, VO_2_ at lactate threshold, and percent VO_2_peak at lactate threshold), and body composition (lean mass (LM) and fat mass (FM)). The z-scores within each grouping were averaged to receive a general “strength and power”, “aerobic”, and “body composition” score. The fat mass was given a negatively weighted z-score, as higher fat mass would be indicative of poorer body composition. These three variables were then included in a linear multiple regression to compare to our primary dependent variable, occupational performance as determined by time to complete the OTA, and our secondary exertion variables (core temperature, skin temperature, heart rate, ventilatory rate), perceived exertion (RPE), and cognitive performance outcomes (WCST perseverative, non-perseverative, total errors). All data were assessed for normality using the Shapiro–Wilks test and Q-Q plot inspection, scatter plots of the residuals were inspected to assess homoskedasticity, and multicollinearity was assessed using variance inflation factor (VIF) values and correlation coefficients of the predictor variables. VIF values were all less than 3.0 [49]; however, strength and power scores were highly correlated with body composition scores (R^2^ = 0.77). Participants with missing data were not included in the analysis of outcomes that the data were missing from. Data are expressed as mean ± standard deviation. Statistical significance for this investigation was set a priori at *p* ≤ 0.05 for all measurements. R Statistical Programming Software Version 4.1.2 (RStudio, Boston, MA, USA) was utilized for all analyses [50]. The application G*Power (version 3.1) was utilized a priori to determine a sample size of 32 participants to appropriately power this study. The following values were input for the estimation of sample size in G*Power: test family = F tests, linear multiple regression, effect size f^2^ = 0.4, α error probability = 0.05, 1-β = 0.80, and number of predictors = 3. This estimated effect size was a conservative estimate determined based on previous research observing effect sizes of this size with similar outcomes [51,52].

## 3. Results

### 3.1. Demographic Values

Thirty-three of these participants (females/males: 15/18; 25.18 ± 4.06 years; 174.01 ± 9.77 cm; 75.94 ± 13.18 kg) were included in the current investigation. Full details, including mean and standard deviations for the fitness assessments, are included in Table 1. The correlation between the variables in the regression model can be found in Table 2. The other eight participants withdrew due to scheduling conflicts or an inability to complete the exertional components of this study.

### 3.2. Occupational Performance and Perceived Exertion

A multiple regression was run to predict time to complete the OTA from the strength and power, aerobic capacity, and body composition z-scores. This resulted in a significant model (F(3, 28) = 8.973; *p* < 0.001; adj. R^2^ = 0.436). The individual predictors were examined further and indicated that strength and power score was a significant predictor (t = −2.052; *p* = 0.050), but not body composition (t = 1.271; *p* = 0.214) or aerobic capacity scores (t = −1.268; *p* = 0.215). See Table 3 for full model details. A multiple regression was run to predict average RPE during the OTA from strength and power, aerobic capacity, and body composition. This resulted in a significant model (F(3, 29) = 3.569; *p* = 0.027; adj. R^2^ = 0.194). The individual predictors were examined further and indicated that strength and power (t = −2.212; *p* = 0.035) as well as aerobic capacity (t = −2.336; *p* = 0.027) scores were significant predictors, but not body composition (t = 1.582; *p* = 0.125). See Table 4 for full model details.

### 3.3. Physiological Response to OTA

A multiple regression was run to predict average core temperature during the OTA from strength and power, aerobic capacity, and body composition z-scores. This resulted in a nonsignificant model (F(3, 29) = 1.490; *p* = 0.238, adj. R^2^ = 0.044). A multiple regression was run to predict average skin temperature during the OTA from strength and power, aerobic capacity, and body composition scores. This resulted in a nonsignificant model (F(3, 29) = 2.791; *p* = 0.058; adj. R^2^ = 0.144). A multiple regression was run to predict average heart rate during the OTA from strength and power, aerobic capacity, and body composition scores. This resulted in a nonsignificant model (F(3, 29) = 0.416; *p* = 0.743; adj. R^2^ = −0.058). A multiple regression was run to predict average ventilatory rate during the OTA from strength and power, aerobic capacity, and body composition scores. This resulted in a nonsignificant model (F(3, 29) = 1.827; *p* = 0.164; adj. R^2^ = 0.072). A multiple regression was run to predict average blood lactate concentrations during the OTA from strength and power, aerobic capacity, and body composition scores. This resulted in a significant model (F(3, 29) = 5.066; *p* = 0.006; adj. R^2^ = 0.276). The individual predictors were examined further and indicated that aerobic capacity score was a significant predictor (t = −2.374; *p* = 0.0244), but not body composition (t = 1.159; *p* = 0.256) or strength and power scores (t = 1.006; *p* = 0.323). See Table 5 for full model details.

### 3.4. Cognition During OTA

A multiple regression was run to predict average total errors during the OTA from strength and power, aerobic capacity, and body composition z-scores. This resulted in a nonsignificant model (F(3, 29) = 0.148; *p* = 0.930; adj. R^2^ = −0.093). A multiple regression was run to predict average perseverative errors during the OTA from strength and power, aerobic capacity, and body composition. This resulted in a nonsignificant model (F(3, 29) = 0.045; *p* = 0.987; adj. R^2^ = −0.106). A multiple regression was run to predict average non-perseverative errors during the OTA from strength and power, aerobic capacity, and body composition. This resulted in a nonsignificant model (F(3, 29) = 0.587; *p* = 0.629; adj. R^2^ = −0.043). See Table 6 for full model details.

## 4. Discussion

This investigation is the first to comprehensively assess the influence of fitness level on occupationally relevant occupational performance, cognitive performance, and exertion during simulated firefighter occupational tasks. We observed the collective strength and power score was the most effective predictor of occupational performance. Strength and power and aerobic capacity scores were significant predictors of perceived exertion during occupational tasks. Aerobic capacity score was also a significant predictor of blood lactate accumulation. Body composition score was not a significant predictor, above what is explained by strength and power score, for any outcomes in this study. However, this is not because body composition is not a meaningful predictor, but rather strength and power score and body composition score are explaining the same variance due to the tight correlation between the two predictors. The findings of this study will help departmental leadership determine fitness assessments relevant for determining occupational readiness in their firefighters.

Our main findings suggest our model significantly predicted time to complete the simulated occupational tasks and perceived exertion. This model suggests that for an individual one standard deviation above the mean in strength and power score, aerobic capacity score, and body composition score, they would be expected to complete the occupational task assessment 155 s (~2.5 min) faster. The model predicted a larger portion of the variance (42%); however, 58% of the variability in time to complete the occupational task remains unexplained. Strength/power was the only significant predictor of time to complete within the model, while body composition and aerobic capacity were not significant predictors within the model. For a one standard deviation increase in strength and power score, we expect occupational task assessment time to decrease by ~126 s (~2 min). This suggests that, when strength and power are assessed, the aerobic capacity and body composition measures tested in this investigation were not effective in providing additional predictive insight into an individual’s ability to complete the occupational task assessment. The relationship between strength and power score and time to complete occupational tasks is consistent with previous literature [53,54]. However, these same studies suggest aerobic endurance significantly predicted higher work rates and faster time to complete simulated firefighting tasks [53,54]. This divergence from our findings may relate to the occupational tasks chosen, in which hose drags, breaching, forcible entry, search, and rescue compare to our lift and carry tasks. Taking the findings from both studies may highlight situational fitness demands. The occupational tasks in both our investigation and previous research are common tasks performed by firefighters. However, the occupational tasks performed by a firefighter can drastically vary given the type of emergency, the geographical region, and staffing. For example, the lift and carry tasks in this investigation were designed to reflect common tasks required of firefighters during non-structural fire emergencies, which make up a majority of calls for firefighters in the southeastern United States [28]. Previous work using hose drags, breaches, and search and rescue tasks may more accurately reflect the physical fitness parameters needed by firefighters during a structure fire, which are critical for firefighters to be physically prepared for [53,54]. Therefore, a combination of these results may provide the most comprehensive fitness needs of a firefighter. Methodological differences between these studies could also contribute to the differences in results seen. The prior studies assessed aerobic endurance using the 30-15 Intermittent Fitness Test [55] and modified Canadian Aerobic Fitness Test [56], while our study used the aerobic capacity score (combination of the modified Bruce protocol [57] to obtain VO_2_peak and lactate threshold). This may suggest that individual field-based assessments of aerobic endurance may translate to the occupational aerobic demands of a firefighter more than laboratory assessments. However, because occupational tasks differed between the investigations, future research should directly compare these assessments to determine the most effective assessment for occupationally relevant aerobic capacity.

Our model was able to significantly predict the RPE experienced during the occupational tasks. This model suggests that for an individual with a strength and power score, aerobic capacity score, and body composition score one standard deviation above the mean, we would predict their perceived exertion would be 1.1 units lower on the RPE scale. This model explained 20% of the variance in perceived exertion; however, many of the factors influencing RPE remain unclear. Further investigation highlighted that when considered together, strength and power as well as aerobic capacity scores were significant predictors of RPE, but not the body composition score. For a one standard deviation increase in either strength and power score or aerobic capacity score, we expect a ~1 unit decrease in RPE. Therefore, aerobic capacity may not be a significant predictor of the speed of occupational task performance but may relate to the effort experienced. This finding becomes most relevant when considering less time-sensitive occupational demands that are extensive or tedious in nature, such as salvage and overhaul, where firefighters are tasked with protecting property and belongings from damage as well as ensuring any remaining sub-fires are completely extinguished [58,59]. A lower RPE could suggest a lower relative intensity that subsequently allows the individual to perform occupational tasks for an extended duration compared to their less strong and aerobically fit counterparts. Body composition score did not help to explain the individual’s perceived effort during the occupational tasks, above what was explained by strength and power score and aerobic capacity score. This, along with our correlation matrix, suggests perceived exertion during occupational tasks may be more related to an individual’s fitness level (strength and power R^2^ = 0.26; aerobic capacity R^2^ = 0.39) rather than their body composition (R^2^ = 0.08).

As a set, strength and power, aerobic capacity, and body composition scores were not predictive of core temperature, skin temperature, heart rate, or ventilatory rate during the occupational task assessment. The assessment’s best-effort nature (rather than a standard rate of exertion) likely led to similar relative working intensities but faster time to complete, which would produce similar physiological responses across participants of different fitness levels. The similarity of responses across participants would leave relatively little variation in the participant’s response during the OTA to be explained by the predictor variables. It is worth noting that while not statistically significant, the model explained ~14% of the variance in skin temperature response, and a one standard deviation change in strength and power was associated with a decrease in skin temperature by ~0.4 °C. This may have practically meaningful differences in physiological and behavioral thermoregulatory responses during firefighter occupational tasks.

Additionally, our model significantly predicted post-occupational blood lactate concentrations. This model suggests that for an individual with a strength and power score, aerobic capacity score, and body composition score that is one standard deviation above the mean, we would expect a blood lactate concentration 0.48 mMol higher following an occupational task assessment. Our model explained ~28% of the variance in blood lactate concentrations during occupational tasks. Specifically, aerobic capacity score was a significant predictor. For a one standard deviation increase in aerobic capacity score, we see a 2.08 mMol decrease in blood lactate concentrations immediately following an occupational task assessment. This is as expected, as the aerobic capacity outcomes include the percentage of VO_2_peak at lactate threshold. Those with a higher percentage of VO_2_peak at lactate threshold were likely able to work at a higher percentage of max intensity without dramatic rises in lactate [60,61]. Accumulation of lactate is associated with acidosis, which can eventually result in fatigue and impair occupational performance [51]. Previous research highlighting the relationship between blood lactate concentration and rating of perceived exertion [62] provides a potential rationale for fitness to influence RPE despite the lack of differences in other exertion variables. Therefore, even though the aerobic capacity score did not predict time to complete the task, it suggested lower lactate accumulation following occupational tasks, which, over extended operations, may enable a firefighter to maintain occupational performance with less fatigue and a lower perception of exertion.

Contrary to our hypothesis, these fitness parameters provided negligible predictive insight into an individual’s cognitive performance during occupational tasks. The model suggests that for a one standard deviation increase in strength and power score, aerobic capacity score, or body composition score, an individual would see less than 0.5 change in total, perseverative, or non-perseverative errors. This is defined as a negligible difference when considering that out of 60 trials, that difference equates to less than a 1% change in performance. No study, to our knowledge, has looked at the influence of fitness on cognitive function during firefighter occupational tasks. However, the related literature has shown high levels of fitness to be associated with better cognitive function [31]. This guided our hypothesis that cognitive flexibility would improve based on fitness level. Previous research has also suggested that exercise can act to prevent declines in cognitive function with aging [63,64,65] and a sedentary lifestyle [66]. It is possible that the overall young and healthy participants in this study limited our ability to see the influence of fitness on cognitive flexibility. Future work in this area should interrogate the relationship between age and activity levels on cognitive function in fire services. This may also suggest that fitness plays a negligible role in cognitive performance during occupational tasks and therefore may require fire departments to use a separate assessment to determine cognitive readiness for firefighter occupational demands.

Additionally, in opposition to our hypothesis, body composition did not significantly increase the predictive power of our models. However, as highlighted by the correlation matrix in Table 2, body composition score and strength and power score were tightly correlated in our study, suggesting that body composition score is explaining much of the same variance that is explained by strength and power score. When considering this and our model results, we see that body composition may be a significant predictor, but not above or beyond that of strength and power scores. Without neglecting the protective health benefits and quality of life factors related to body composition, these findings may support the notion that when fitness is assessed, body composition does not provide additional insight related to a firefighter’s occupational performance. This is in line with previous work that supports that fitness is also more predictive than body composition for general health and mortality risk [67,68]. Previous research has shown that body composition is a significant predictor of occupational performance in fire services [53,69]. Taken together, this suggests that body composition alone can predict performance, but fitness measures (i.e., strength, power, or aerobic capacity) may be a more robust predictor of many occupationally relevant outcomes.

One limitation of this study is the computerized, controlled configuration of the WCST may not be indicative of the exact cognitive demands placed on firefighters in occupational scenarios. The WCST was chosen in an effort to ensure a validated assessment of cognitive flexibility; however, future studies should work to develop and validate tactically relevant assessments of cognitive function that provide validity and practicality. The current investigation also included local community members rather than career or volunteer firefighters. While previous literature suggests that firefighters are on average older and have poorer cardiovascular outcomes [70], the local fire departments in our region are younger [11] and report being more physically active [28] than the national average and therefore align closely with the age and activity level of the population used in this investigation. Therefore, when considering this and the limited number of local firefighters and, more specifically, female firefighters, the use of a local community ensured an adequate and diverse sample population. However, we acknowledge that long-term career firefighters may have adaptations that result in different physiological responses to occupational tasks [71]; although, the population used in this study could especially aid in understanding appropriate screening methods for new fire-service recruits. As highlighted above, the tasks in this study were chosen to emphasize lift and carry tasks, but there are other tasks, such as breaching or dragging, that could be more or less related to these fitness and health outcomes.

Additionally, our power analysis indicates we are appropriately powered to observe effect sizes of 0.4 or greater, and it is possible that with a larger sample, smaller effects could be observed. Lastly, our participants were all moderately active (exercising 3+ days/week for 30+ min/day), and future research could investigate the impact of fitness on these outcomes in a more diverse sample of fitness levels (i.e., sedentary or highly active individuals).

Collectively, these results provide additional insight that can aid firefighters and their leadership in determining how to train, prepare, and assess relevant metrics of fitness that support occupational performance. The implementation of strength- and power-based assessments by departmental leadership may be the most beneficial fitness assessments to add to candidate physical ability tests (CPATs). Additionally, firefighter fitness programs with a strong emphasis on strength and power may support occupational performance. This is not to suggest that aerobic fitness should not be assessed by departments or be integrated into fitness programs for firefighters, as previous literature has highlighted the importance of aerobic fitness from an occupational standpoint [53,54]. Specifically, firefighters have a high prevalence of cardiovascular disease and comorbidities [72,73] that make regular aerobic exercise imperative to their overall health and longevity [19]. Additionally, as firefighters are frequently exposed to extreme environmental conditions, improvements in aerobic fitness can improve heat tolerance and reduce the risk of exertional heat illness [74].

## 5. Conclusions

We observed that when taken together, aerobic capacity, body composition, strength, and power assessments may be useful measures to predict a firefighter’s physical performance and perceived exertion during occupational tasks. However, within the set of variables, strength and power appear to be robust predictors of occupational task performance, in which aerobic capacity and body composition did not provide additional predictive ability. Aerobic capacity, strength, and power appear to be robust predictors of perceived exertion. Therefore, the assessment of body composition does not provide additional insight into occupational readiness when strength and power are assessed. These outcomes may also be useful in determining some metrics of exertion experienced during simulated occupational tasks. However, we did not observe a utility for assessing fitness in determining cognitive performance during occupational tasks. Fire departments can use these findings to guide the assessments used for screening occupational readiness in firefighters and recruits.

## Figures and Tables

**Table 1:** Participant performance demographics.

Variable	Mean ± SD
Strength and Power	
Composite strength and power z-scores	0.00 ± 0.92
Barbell bench 3 repetition maximum lift	78.25 ± 35.97 kg
Barbell back squat 3 repetition maximum lift	106.85 ± 34.07 kg
Hex-bar deadlift 3 repetition maximum lift	120.34 ± 42.12 kg
Maximum vertical jump height	52.86 ± 12.80 cm
Aerobic Capacity	
Composite aerobic capacity z-scores	0.00 ± 0.71
VO_2_peak	46.26 ± 7.34 mL/kg/min
VO_2_ at lactate threshold	29.51 ± 6.23 mL/kg/min
Percent VO_2_peak at lactate threshold	64.51 ± 12.83%
Body Composition	
Composite body composition z-scores	0.00 ± 0.78
Lean mass	57.21 ± 12.96 kg
Fat mass	16.56 ± 5.67 kg

SD = standard deviation. Note: Composition scores are the mean and standard deviation of a z-score; therefore, the means will always equal 0. A mean of 0—z-score would represent an individual having the listed average individual performance values.

**Table 2:** Correlation Matrix.

	AC	BC	SP	TTC	RPE	BLC	CT	ST	HR	VE
AC	1.00									
BC	0.16	1.00								
SP	0.04	0.77	1.00							
TTC	0.13	−0.59	−0.62	1.00						
RPE	−0.39	−0.08	−0.26	−0.21	1.00					
BLC	−0.34	0.37	0.39	−0.59	0.24	1.00				
CT	−0.25	−0.25	−0.09	0.01	0.35	0.35	1.00			
ST	0.17	−0.03	−0.02	0.34	0.11	−0.45	−0.07	1.00		
HR	−0.15	−0.10	0.10	−0.25	0.33	0.56	0.77	−0.41	1.00	
VR	−0.42	0.07	−0.29	−0.49	0.31	0.45	0.10	−0.42	0.42	1.00
TE	−0.04	−0.01	−0.08	−0.32	−0.03	0.03	−0.17	0.04	0.05	0.19
PE	0.02	0.06	0.02	−0.37	0.02	0.17	−0.14	−0.07	0.16	0.24
NPE	0.17	−0.17	−0.18	−0.12	−0.05	−0.11	−0.12	0.07	−0.03	0.08

AC: aerobic capacity score; BC: body composition score; SP: strength and power score; TTC: total time to complete; RPE: rating of perceived exertion (Borg 6–20 scale); BLC: blood lactate concentrations (mMol); CT: core temperature (°C); ST: skin temperature (°C); HR: heart rate (beats/min); VR: ventilatory rate (breaths/min); TE: total errors; PE: perseverative errors; NPE: non-perseverative errors.

**Table 3:** Model prediction for time to complete OTA.

	Estimate	Std. Error
Intercept	1125.13 s	34.91 s
Strength and power z-score	−126.71 * s	61.75 s
Aerobic z-score	64.36 s	50.63 s
Body composition z-score	−93.30 s	73.57 s

OTA = occupational task assessment; Std. = standard; * denotes *p* < 0.05.

**Table 4:** Model prediction for RPE during OTA.

	Estimate	Std. Error
Intercept	12.72	0.28
Strength and power z-score	−1.08 *	0.49
Aerobic z-score	−0.95 *	0.41
Body composition z-score	0.92	0.58

RPE = rating of perceived exertion (Borg 6–20 scale); OTA = occupational task assessment; Std. = standard; * denotes *p* < 0.05.

**Table 5:** Model prediction for exertion during OTA.

	Intercept		Strength and Power Z-Score		Aerobic Capacity Z-Score		Body Composition Z-Score	
Variables	Estimate	SE	Estimate	SE	Estimate	SE	Estimate	SE
Core Temp (°C)	37.55	0.06	0.05	0.11	−0.10	0.09	−0.16	0.13
Skin Temp (°C)	36.32	0.09	−0.42 *	0.16	0.01	0.13	0.32	0.18
Heart rate (bpm)	148.40	2.86	2.90	5.13	−2.70	4.21	−4.64	6.12
Ventilatory rate (brpm)	37.63	0.90	0.61	1.61	−2.77	1.32	0.79	1.92
BLC (mMol)	8.03	0.60	1.08	1.07	−2.08 *	0.88	1.48	1.27

OTA = occupational task assessment; Temp = temperature; bpm = beats/minute; brpm = breaths/minute; BLC = blood lactate concentrations; mMol = millimolar; * denotes *p* < 0.05.

**Table 6:** Model prediction for cognitive flexibility during OTA.

	Intercept		Strength and Power Z-Score		Aerobic Capacity Z-Score		Body Composition Z-Score	
Variables	Estimate	SE	Estimate	SE	Estimate	SE	Estimate	SE
Total errors	8.23	0.43	−0.49	0.78	−0.18	0.62	0.42	0.90
Perseverative errors	6.03	0.27	−0.07	0.49	0.01	0.38	0.18	0.56
Non-perseverative errors	2.26	0.29	−0.27	0.51	−0.35	0.41	−0.05	0.59

OTA = occupational task assessment; SE = standard error.

## Data Availability

Data can be found at https://figshare.com/articles/dataset/Agostinelli_Dissertation_Aim3/28768673/1?file=53577977.
